# Visualization and Quantification of Regional Ventricular Wall and Blood Velocities by 3T 4D PC-MRI

**DOI:** 10.1186/1532-429X-18-S1-P349

**Published:** 2016-01-27

**Authors:** Stig F Samnøy, Gottfried Greve, Kirsti Lysaker, Terje H Larsen

**Affiliations:** 1 grid.457585.e0000000417549964Bergen University College, Bergen, Norway; 2 grid.7914.b0000000419367443Department of Clinical Science, University of Bergen, Bergen, Norway; 3 Department of Heart Disease, Unive, Bergen, Norway

## Background

Wall motion together with the development of intra-cavity pressure and inside shear stress cause synchronous myocardial contraction and relaxation, as well as blood flow. These variables are related to cardiovascular function and disease. Assessing regional wall velocities and blood flow may therefore provide quantitative information regarding abnormalities in wall contractility and alterations in blood flow patterns. By using a retrospective 4D PC-MRI technique, this study aimed to develop a tool to simultaneously visualize and quantify both regional ventricular wall velocity and the intra-cavity blood flow pattern.

## Methods

Consecutive 2D short-axis slices with three orthogonal velocity components covering the whole heart throughout the cardiac cycle were collected using a retrospective 4D PC-MRI, ECG gated CINE imaging technique during breath-hold. The scanner for the acquisitions was a 3.0T GE Signa Excite scanner (Milwaukee, WI, USA), and sequence parameters were TR = 11 ms, TE = 4 ms, Flip Angle = 20°, Matrix = 256 × 256, Slice Thickness = 8 mm, Slice Resolution = 1.25 mm per pixel, VENC = 150 cm/s, Field of View = 320 × 320 mm.

Ventricular walls were extracted from the temporal images in all slices and pixel positions of the time dependent walls formed the basis for a surface model of the ventricles. The wall-surface was colored according to the resultant velocities. The intra-cavity blood flow was visualized using 3D vectors in each pixel position. Both the wall and blood flow pattern were studied simultaneously in a fully rotatable and scalable 3D frame.

## Results

**Figure 1:**
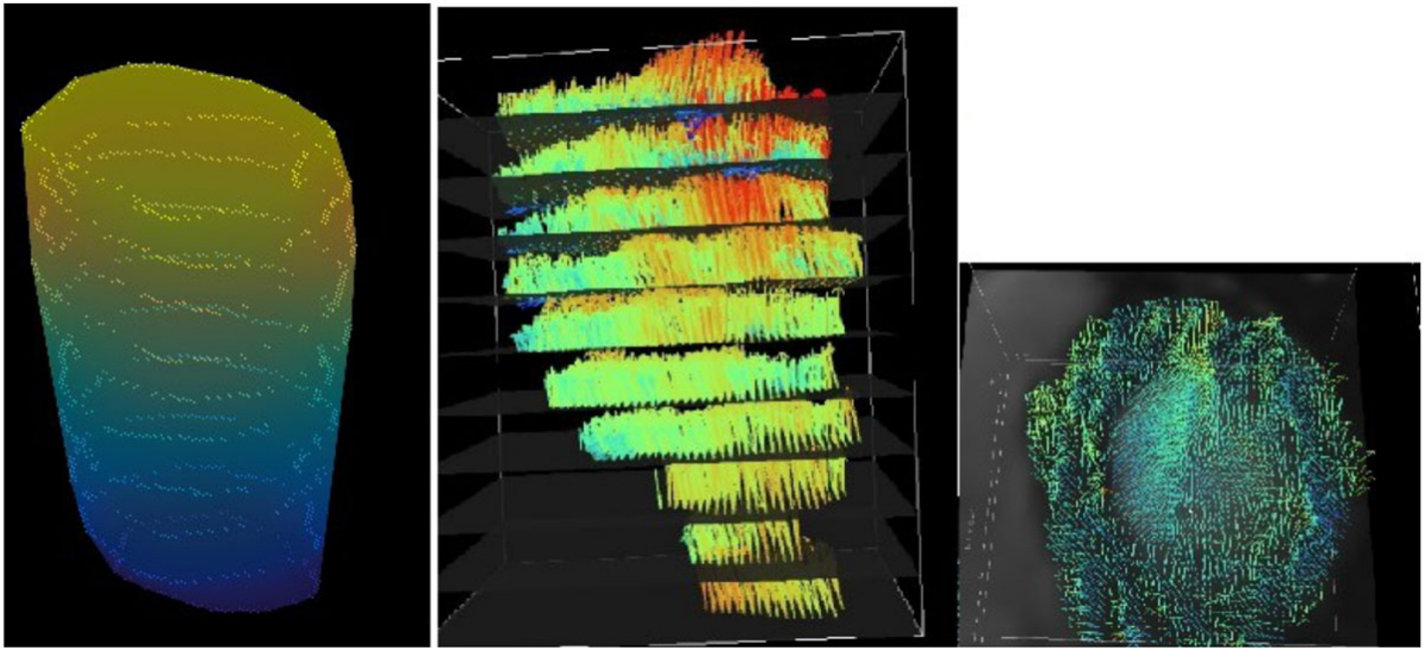
Figure 1

By applying a retrospective 4D PC-MRI technique on the whole heart our software was able to present velocities of the ventricular wall as a color-coded surface, and the corresponding intra-cavity blood velocity pattern as velocity vectors in the same visual model. This was performed using data for the whole heart including both left and right ventricle from healthy volunteers. Figure 1 illustrates left ventricular wall and blood velocities at different time samples.

## Conclusions

This method improves the ability to simultaneously study global and regional velocity patterns of the ventricular walls and the corresponding blood velocity pattern. This technique may reveal hypokinetic and akinetic contractions, as well as asynchronous and paradox wall movements and abnormal velocity flow patterns.

